# 1,2-Bis(di-2-pyridylphosphino­yl)ethane

**DOI:** 10.1107/S1600536809004590

**Published:** 2009-02-18

**Authors:** Susan J. Berners-Price, Maribel Navarro, Brian W. Skelton

**Affiliations:** a Chemistry, School of Biomedical, Biomolecular and Chemical Sciences, The University of Western Australia, 35 Stirling Highway, Crawley, Perth 6009, Western Australia, Australia

## Abstract

The crystal structure of the title compound, C_22_H_20_N_4_O_2_P_2_, consists of two independent half-mol­ecules, both of which lie on crystallographic inversion centres. There are no significant differences between the two mol­ecules.

## Related literature

For the anti­tumour properties of metal complexes of bidentate tertiary phosphine ligands with pyridyl substituents, see: McKeage *et al.* (2000 ▶); Barnard & Berners-Price (2007 ▶); Liu *et al.* (2008 ▶). The crystal structure of the parent 1,2-bis­(di-2-pyridylphosphino)ethane mol­ecule has been determined (Jones *et al.*, 1999 ▶). The structure of 1,2-bis­(di-phenyl­phosphino)ethane dioxide (Calcagno *et al.*, 2000 ▶) is similar, with the two halves of the mol­ecule related by a pseudo-inversion centre, but this is not isomorphous with the title compound.
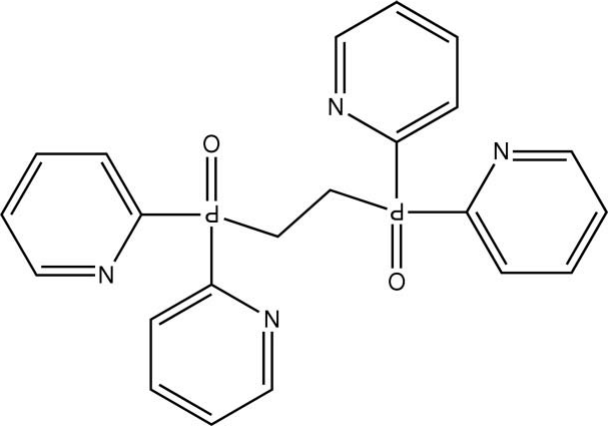


## Experimental

### 

#### Crystal data

C_22_H_20_N_4_O_2_P_2_
*M* *_r_* = 434.36
Triclinic, 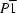

*a* = 8.3760 (6) Å
*b* = 8.8496 (8) Å
*c* = 16.2332 (11) Å
α = 105.627 (7)°
β = 92.429 (5)°
γ = 112.559 (7)°
*V* = 1055.67 (16) Å^3^
*Z* = 2
Mo *K*α radiation
μ = 0.23 mm^−1^
*T* = 110 K
0.22 × 0.10 × 0.06 mm

#### Data collection

Oxford Diffraction Gemini diffractometer
Absorption correction: Gaussian (**CrysAlis RED**; Oxford Diffraction, 2008 ▶) *T*_min_ = 0.968, *T*_max_ = 0.988
10983 measured reflections
4842 independent reflections
2698 reflections with *I* > 2σ(*I*)
*R*_int_ = 0.059

#### Refinement

*R*[*F* ^2^ > 2σ(*F* ^2^)] = 0.052
*wR*(*F* ^2^) = 0.112
*S* = 0.86
4842 reflections
271 parameters
H-atom parameters constrained
Δρ_max_ = 0.43 e Å^−3^
Δρ_min_ = −0.34 e Å^−3^

### 

Data collection: *CrysAlis CCD* (Oxford Diffraction, 2008 ▶); cell refinement: *CrysAlis RED* (Oxford Diffraction, 2008 ▶); data reduction: *CrysAlis RED*; program(s) used to solve structure: *SIR92* (Altomare *et al.*, 1994 ▶); program(s) used to refine structure: *SHELXL97* (Sheldrick, 2008 ▶); molecular graphics: *ORTEPII* (Johnson, 1976 ▶); software used to prepare material for publication: *publCIF* (Westrip, 2009 ▶).

## Supplementary Material

Crystal structure: contains datablocks I, global. DOI: 10.1107/S1600536809004590/fj2193sup1.cif

Structure factors: contains datablocks I. DOI: 10.1107/S1600536809004590/fj2193Isup2.hkl

Additional supplementary materials:  crystallographic information; 3D view; checkCIF report

## Figures and Tables

**Fig. 1.**
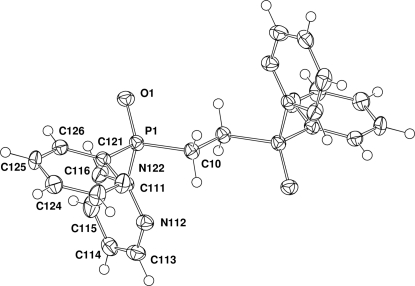
ORTEP drawing and atom labelling for molecule n = 1. Displacement ellipsoids of non-H atoms are drawn at the 50% probablility level. The structure of the second, n = 2, molecule is very similar.

## supplementary crystallographic information

### Comment

Bidentate tertiary phosphine ligands with pyridyl substituents, such as 1,2-bis(di-2-pyridylphosphino)ethane (d2pype) are of interest because a number of studies have shown that metal complexes with these ligands exhibit selective anti-tumour properties (McKeage *et al.*, 2000; Barnard and Berners-Price 2007; Liu *et al.*, 2008). During the course of our work in this area, we obtained crystals of the phosphine oxide d2pypeO_2_ (I), which were suitable for X-ray diffraction studies.

### Experimental

1,2-bis(di-2-pyridylphosphino)ethane (d2pype) was obtained from Strem Chemicals Inc. Single crystals of the title compound d2pypeO_2_ (I) suitable for X-ray crystallographic analysis were obtained as a by-product of slow evaporation of a solution of d2pype and copper (I) iodide (molar ratio 2:1) in acetonitrile-tetrahydrofuran mixture.

### Refinement

The assignments of the py ring N,*C* atoms were made on the basis of refinement and location of the H atoms. All H atoms were positioned geometrically and refined using a riding model with C—H = 0.95–0.99 Å and with *U*_iso_(H) = 1.2 times *U*_eq_(C).

### Crystal data

**Table:**

C_22_H_20_N_4_O_2_P_2_	*Z* = 2
*M**_r_* = 434.36	*F*(000) = 452
Triclinic, *P*1	*D*_x_ = 1.366 Mg m^−^^3^
Hall symbol: -p 1	Mo *K*α radiation, λ = 0.71073 Å
*a* = 8.3760 (6) Å	Cell parameters from 2539 reflections
*b* = 8.8496 (8) Å	θ = 3.3–32.6°
*c* = 16.2332 (11) Å	µ = 0.23 mm^−^^1^
α = 105.627 (7)°	*T* = 110 K
β = 92.429 (5)°	Plate, colourless
γ = 112.559 (7)°	0.22 × 0.10 × 0.06 mm
*V* = 1055.67 (16) Å^3^	

### Data collection

**Table:**

Oxford Diffraction Gemini diffractometer	4842 independent reflections
Radiation source: sealed tube	2698 reflections with *I* > 2σ(*I*)
graphite	*R*_int_ = 0.059
ω scans	θ_max_ = 27.5°, θ_min_ = 3.3°
Absorption correction: gaussian (*CrysAlis RED*; Oxford Diffraction, 2008)	*h* = −10→10
*T*_min_ = 0.968, *T*_max_ = 0.988	*k* = −11→11
10983 measured reflections	*l* = −21→21

### Refinement

**Table:**

Refinement on *F*^2^	Primary atom site location: structure-invariant direct methods
Least-squares matrix: full	Secondary atom site location: difference Fourier map
*R*[*F*^2^ > 2σ(*F*^2^)] = 0.052	Hydrogen site location: inferred from neighbouring sites
*wR*(*F*^2^) = 0.112	H-atom parameters constrained
*S* = 0.86	*w* = 1/[σ^2^(*F*_o_^2^) + (0.0495*P*)^2^] where *P* = (*F*_o_^2^ + 2*F*_c_^2^)/3
4842 reflections	(Δ/σ)_max_ = 0.002
271 parameters	Δρ_max_ = 0.43 e Å^−^^3^
0 restraints	Δρ_min_ = −0.34 e Å^−^^3^

### Special details

**Table:**

Geometry. All e.s.d.'s (except the e.s.d. in the dihedral angle between two l.s. planes) are estimated using the full covariance matrix. The cell e.s.d.'s are taken into account individually in the estimation of e.s.d.'s in distances, angles and torsion angles; correlations between e.s.d.'s in cell parameters are only used when they are defined by crystal symmetry. An approximate (isotropic) treatment of cell e.s.d.'s is used for estimating e.s.d.'s involving l.s. planes.

### Fractional atomic coordinates and isotropic or equivalent isotropic displacement parameters (Å^2^)

**Table:**

	*x*	*y*	*z*	*U*_iso_*/*U*_eq_	
P1	0.18196 (9)	0.21056 (9)	0.45935 (4)	0.02233 (18)	
O1	0.3253 (2)	0.3074 (2)	0.53572 (11)	0.0272 (4)	
C111	0.2511 (3)	0.0876 (3)	0.37091 (16)	0.0217 (6)	
N112	0.1239 (3)	−0.0366 (3)	0.30732 (14)	0.0267 (5)	
C113	0.1759 (4)	−0.1218 (4)	0.24034 (17)	0.0320 (7)	
H113	0.0879	−0.2109	0.195	0.038*	
C114	0.3487 (4)	−0.0891 (4)	0.23254 (18)	0.0320 (7)	
H114	0.3782	−0.1528	0.1831	0.038*	
C115	0.4767 (4)	0.0385 (4)	0.29857 (19)	0.0372 (8)	
H115	0.5969	0.0641	0.2957	0.045*	
C116	0.4284 (3)	0.1287 (4)	0.36902 (18)	0.0313 (7)	
H116	0.5146	0.2172	0.4153	0.038*	
C121	0.1197 (3)	0.3509 (3)	0.41523 (15)	0.0227 (6)	
N122	−0.0518 (3)	0.2959 (3)	0.38395 (15)	0.0300 (6)	
C123	−0.0959 (4)	0.4009 (4)	0.35135 (19)	0.0352 (7)	
H123	−0.216	0.3661	0.3298	0.042*	
C124	0.0236 (4)	0.5571 (4)	0.34727 (17)	0.0310 (7)	
H124	−0.0142	0.6263	0.3228	0.037*	
C125	0.1972 (4)	0.6111 (4)	0.37892 (17)	0.0316 (7)	
H125	0.2816	0.7184	0.3773	0.038*	
C126	0.2464 (3)	0.5055 (3)	0.41316 (16)	0.0256 (6)	
H126	0.3659	0.5388	0.4351	0.031*	
C10	−0.0180 (3)	0.0583 (3)	0.47773 (16)	0.0242 (6)	
H10A	−0.0786	0.121	0.5142	0.029*	
H10B	−0.0962	−0.0124	0.4216	0.029*	
P2	0.68020 (8)	0.27338 (9)	0.04404 (4)	0.02132 (18)	
O2	0.7095 (2)	0.3187 (2)	−0.03767 (11)	0.0278 (4)	
C211	0.6499 (3)	0.4398 (3)	0.12649 (16)	0.0213 (6)	
N212	0.6135 (3)	0.4071 (3)	0.20118 (14)	0.0279 (5)	
C213	0.6005 (4)	0.5335 (4)	0.26420 (18)	0.0327 (7)	
H213	0.5758	0.5138	0.3179	0.039*	
C214	0.6210 (3)	0.6903 (4)	0.2556 (2)	0.0358 (7)	
H214	0.6111	0.7759	0.3025	0.043*	
C215	0.6559 (3)	0.7211 (4)	0.1781 (2)	0.0347 (7)	
H215	0.6695	0.8277	0.1702	0.042*	
C216	0.6707 (3)	0.5931 (4)	0.11223 (19)	0.0299 (7)	
H216	0.6949	0.61	0.058	0.036*	
C221	0.8672 (3)	0.2545 (3)	0.09411 (16)	0.0218 (6)	
N222	0.8365 (3)	0.1354 (3)	0.13511 (14)	0.0285 (5)	
C223	0.9776 (4)	0.1286 (4)	0.17259 (18)	0.0335 (7)	
H223	0.9594	0.0457	0.2022	0.04*	
C224	1.1484 (4)	0.2342 (4)	0.17114 (18)	0.0330 (7)	
H224	1.2439	0.2227	0.1984	0.04*	
C225	1.1771 (3)	0.3559 (4)	0.12949 (18)	0.0342 (7)	
H225	1.2929	0.4316	0.1281	0.041*	
C226	1.0340 (3)	0.3661 (4)	0.08959 (17)	0.0278 (6)	
H226	1.0498	0.4481	0.0596	0.033*	
C20	0.4938 (3)	0.0766 (3)	0.03363 (16)	0.0212 (6)	
H20A	0.4874	0.0554	0.0905	0.025*	
H20B	0.3853	0.0876	0.0159	0.025*	

### Atomic displacement parameters (Å^2^)

**Table:**

	*U*^11^	*U*^22^	*U*^33^	*U*^12^	*U*^13^	*U*^23^
P1	0.0206 (4)	0.0253 (4)	0.0224 (4)	0.0096 (3)	0.0051 (3)	0.0090 (3)
O1	0.0232 (9)	0.0318 (11)	0.0235 (10)	0.0099 (9)	0.0022 (8)	0.0063 (8)
C111	0.0238 (14)	0.0239 (15)	0.0205 (13)	0.0105 (12)	0.0034 (11)	0.0107 (12)
N112	0.0328 (13)	0.0261 (14)	0.0234 (12)	0.0148 (11)	0.0026 (10)	0.0073 (11)
C113	0.0433 (18)	0.0298 (17)	0.0235 (15)	0.0168 (15)	0.0002 (13)	0.0072 (13)
C114	0.0463 (18)	0.0262 (17)	0.0295 (16)	0.0186 (15)	0.0148 (14)	0.0115 (14)
C115	0.0328 (16)	0.0311 (18)	0.050 (2)	0.0137 (14)	0.0224 (15)	0.0120 (16)
C116	0.0261 (15)	0.0256 (17)	0.0358 (17)	0.0058 (13)	0.0070 (13)	0.0063 (14)
C121	0.0250 (14)	0.0237 (15)	0.0201 (14)	0.0105 (12)	0.0067 (11)	0.0066 (12)
N122	0.0216 (12)	0.0288 (14)	0.0420 (14)	0.0083 (11)	0.0007 (10)	0.0181 (12)
C123	0.0262 (15)	0.0337 (19)	0.0480 (19)	0.0103 (14)	0.0022 (13)	0.0197 (15)
C124	0.0354 (16)	0.0282 (17)	0.0341 (16)	0.0159 (14)	0.0042 (13)	0.0128 (14)
C125	0.0342 (16)	0.0201 (16)	0.0361 (17)	0.0037 (13)	0.0084 (13)	0.0126 (13)
C126	0.0239 (14)	0.0268 (16)	0.0232 (14)	0.0083 (12)	0.0031 (11)	0.0065 (12)
C10	0.0213 (14)	0.0279 (16)	0.0256 (14)	0.0110 (12)	0.0061 (11)	0.0104 (12)
P2	0.0193 (3)	0.0198 (4)	0.0227 (4)	0.0056 (3)	0.0010 (3)	0.0071 (3)
O2	0.0289 (10)	0.0251 (11)	0.0265 (10)	0.0079 (9)	0.0018 (8)	0.0084 (9)
C211	0.0125 (12)	0.0214 (15)	0.0253 (14)	0.0052 (11)	−0.0033 (11)	0.0037 (12)
N212	0.0272 (12)	0.0358 (15)	0.0220 (12)	0.0161 (11)	−0.0015 (10)	0.0070 (11)
C213	0.0332 (16)	0.044 (2)	0.0234 (15)	0.0229 (15)	0.0014 (12)	0.0042 (14)
C214	0.0262 (15)	0.0336 (19)	0.0426 (19)	0.0162 (14)	0.0004 (14)	−0.0008 (15)
C215	0.0232 (15)	0.0180 (16)	0.057 (2)	0.0052 (12)	0.0057 (14)	0.0069 (15)
C216	0.0195 (14)	0.0267 (17)	0.0400 (17)	0.0058 (12)	0.0060 (12)	0.0104 (14)
C221	0.0225 (14)	0.0214 (15)	0.0208 (14)	0.0089 (12)	0.0039 (11)	0.0053 (12)
N222	0.0250 (12)	0.0265 (14)	0.0348 (13)	0.0087 (11)	0.0012 (10)	0.0141 (11)
C223	0.0295 (16)	0.0307 (18)	0.0421 (18)	0.0093 (14)	0.0003 (13)	0.0191 (15)
C224	0.0234 (15)	0.0388 (19)	0.0399 (18)	0.0134 (14)	−0.0027 (13)	0.0169 (15)
C225	0.0196 (14)	0.0389 (19)	0.0412 (18)	0.0069 (13)	0.0058 (13)	0.0155 (15)
C226	0.0240 (14)	0.0279 (17)	0.0321 (16)	0.0069 (13)	0.0059 (12)	0.0157 (13)
C20	0.0185 (13)	0.0212 (15)	0.0226 (14)	0.0072 (11)	−0.0002 (11)	0.0067 (11)

### Geometric parameters (Å, °)

**Table:**

P1—O1	1.4917 (18)	P2—O2	1.4897 (18)
P1—C10	1.799 (3)	P2—C20	1.798 (2)
P1—C121	1.809 (3)	P2—C211	1.811 (3)
P1—C111	1.815 (3)	P2—C221	1.819 (3)
C111—N112	1.344 (3)	C211—N212	1.341 (3)
C111—C116	1.391 (3)	C211—C216	1.384 (4)
N112—C113	1.339 (3)	N212—C213	1.340 (3)
C113—C114	1.381 (4)	C213—C214	1.378 (4)
C113—H113	0.95	C213—H213	0.95
C114—C115	1.377 (4)	C214—C215	1.378 (4)
C114—H114	0.95	C214—H214	0.95
C115—C116	1.380 (4)	C215—C216	1.382 (4)
C115—H115	0.95	C215—H215	0.95
C116—H116	0.95	C216—H216	0.95
C121—N122	1.352 (3)	C221—N222	1.343 (3)
C121—C126	1.383 (4)	C221—C226	1.386 (3)
N122—C123	1.339 (3)	N222—C223	1.336 (3)
C123—C124	1.382 (4)	C223—C224	1.382 (4)
C123—H123	0.95	C223—H223	0.95
C124—C125	1.371 (4)	C224—C225	1.372 (4)
C124—H124	0.95	C224—H224	0.95
C125—C126	1.381 (4)	C225—C226	1.383 (4)
C125—H125	0.95	C225—H225	0.95
C126—H126	0.95	C226—H226	0.95
C10—C10^i^	1.516 (5)	C20—C20^ii^	1.536 (5)
C10—H10A	0.99	C20—H20A	0.99
C10—H10B	0.99	C20—H20B	0.99
			
O1—P1—C10	115.87 (11)	O2—P2—C20	115.28 (11)
O1—P1—C121	112.56 (12)	O2—P2—C211	111.60 (12)
C10—P1—C121	105.88 (12)	C20—P2—C211	106.06 (11)
O1—P1—C111	110.98 (11)	O2—P2—C221	112.89 (11)
C10—P1—C111	105.53 (12)	C20—P2—C221	106.09 (12)
C121—P1—C111	105.23 (11)	C211—P2—C221	104.05 (11)
N112—C111—C116	123.1 (2)	N212—C211—C216	123.4 (2)
N112—C111—P1	116.64 (18)	N212—C211—P2	116.2 (2)
C116—C111—P1	120.2 (2)	C216—C211—P2	120.4 (2)
C113—N112—C111	116.4 (2)	C213—N212—C211	116.5 (2)
N112—C113—C114	124.6 (3)	N212—C213—C214	123.8 (3)
N112—C113—H113	117.7	N212—C213—H213	118.1
C114—C113—H113	117.7	C214—C213—H213	118.1
C115—C114—C113	118.0 (3)	C215—C214—C213	119.1 (3)
C115—C114—H114	121	C215—C214—H214	120.5
C113—C114—H114	121	C213—C214—H214	120.5
C114—C115—C116	119.3 (3)	C214—C215—C216	118.2 (3)
C114—C115—H115	120.4	C214—C215—H215	120.9
C116—C115—H115	120.4	C216—C215—H215	120.9
C115—C116—C111	118.7 (3)	C215—C216—C211	119.0 (3)
C115—C116—H116	120.6	C215—C216—H216	120.5
C111—C116—H116	120.6	C211—C216—H216	120.5
N122—C121—C126	123.0 (2)	N222—C221—C226	123.4 (2)
N122—C121—P1	117.2 (2)	N222—C221—P2	118.23 (18)
C126—C121—P1	119.86 (19)	C226—C221—P2	118.36 (19)
C123—N122—C121	116.5 (2)	C223—N222—C221	116.2 (2)
N122—C123—C124	123.6 (3)	N222—C223—C224	124.3 (3)
N122—C123—H123	118.2	N222—C223—H223	117.8
C124—C123—H123	118.2	C224—C223—H223	117.8
C125—C124—C123	119.3 (3)	C225—C224—C223	118.6 (2)
C125—C124—H124	120.4	C225—C224—H224	120.7
C123—C124—H124	120.4	C223—C224—H224	120.7
C124—C125—C126	118.4 (3)	C224—C225—C226	118.7 (3)
C124—C125—H125	120.8	C224—C225—H225	120.6
C126—C125—H125	120.8	C226—C225—H225	120.6
C125—C126—C121	119.3 (2)	C225—C226—C221	118.8 (2)
C125—C126—H126	120.4	C225—C226—H226	120.6
C121—C126—H126	120.4	C221—C226—H226	120.6
C10^i^—C10—P1	111.2 (2)	C20^ii^—C20—P2	111.1 (2)
C10^i^—C10—H10A	109.4	C20^ii^—C20—H20A	109.4
P1—C10—H10A	109.4	P2—C20—H20A	109.4
C10^i^—C10—H10B	109.4	C20^ii^—C20—H20B	109.4
P1—C10—H10B	109.4	P2—C20—H20B	109.4
H10A—C10—H10B	108	H20A—C20—H20B	108

Symmetry codes: (i) −*x*, −*y*, −*z*+1; (ii) −*x*+1, −*y*, −*z*.
